# Enhancing estimation of cover crop biomass using field-based high-throughput phenotyping and machine learning models

**DOI:** 10.3389/fpls.2023.1277672

**Published:** 2024-01-08

**Authors:** Geng Bai, Katja Koehler-Cole, David Scoby, Vesh R. Thapa, Andrea Basche, Yufeng Ge

**Affiliations:** ^1^ Department of Biological Systems Engineering, University of Nebraska-Lincoln, Lincoln, NE, United States; ^2^ Nebraska Extension, University of Nebraska-Lincoln, Ithaca, NE, United States; ^3^ Department of Agronomy and Horticulture, University of Nebraska-Lincoln, Lincoln, NE, United States; ^4^ Center for Plant Science Innovation, University of Nebraska-Lincoln, Lincoln, NE, United States

**Keywords:** aboveground biomass, cover crop, plant phenotyping, machine learning, partial least squares regression, rye

## Abstract

Incorporating cover crops into cropping systems offers numerous potential benefits, including the reduction of soil erosion, suppression of weeds, decreased nitrogen requirements for subsequent crops, and increased carbon sequestration. The aboveground biomass (AGB) of cover crops strongly influences their performance in delivering these benefits. Despite the significance of AGB, a comprehensive field-based high-throughput phenotyping study to quantify AGB of multiple cover crops in the U.S. Midwest has not been found. This study presents a two-year field experiment carried out in Eastern Nebraska, USA, to estimate AGB of five different cover crop species [canola (*Brassica napus* L.), rye (*Secale cereale* L.), triticale (*Triticale × Triticosecale* L.), vetch (*Vicia sativa* L.), and wheat (*Triticum aestivum* L.)] using high-throughput phenotyping and Machine Learning (ML) models. Destructive AGB sampling was performed three times during each spring season in 2022 and 2023. An array of morphological, spectral, thermal, and environmental features from the sensors were utilized as feature inputs of ML models. Moderately strong linear correlations between AGB and the selected features were observed. Four ML models, namely Random Forests Regression (RFR), Support Vector Regression (SVR), Partial Least Squares Regression (PLSR), and Artificial Neural Network (ANN), were investigated. Among the four models, PLSR achieved the highest Coefficient of Determination (R^2^) of 0.84 and the lowest Root Mean Squared Error (RMSE) of 892 kg/ha (Normalized RMSE (NRMSE) = 8.87%), indicating that PLSR could be the most appropriate method for estimating AGB of multiple cover crop species. Feature importance analysis ranked spectral features like Normalized Difference Red Edge (NDRE), Solar-induced Fluorescence (SIF), Spectral Reflectance at 485 nm (R485), and Normalized Difference Vegetation Index (NDVI) as top model features using PLSR. When utilizing fewer feature inputs, ANN exhibited better prediction performance compared to other models. Using morphological and spectral parameters as input features alone led to a R^2^ of 0.80 and 0.77 for AGB prediction using ANN, respectively. This study demonstrated the feasibility of high-throughput phenotyping and ML techniques for accurately estimating AGB of multiple cover crop species. Further enhancement of model performance could be achieved through additional destructive sampling conducted across multiple locations and years.

## 1 Introduction

Cover crops, strategically incorporated into farmland soil during fallow periods between primary cash crop production, serve a multitude of potential benefits. These benefits include the reduction of soil erosion through wind and surface runoff, augmentation of carbon sequestration through biomass accumulation, and the contribution of nitrogen to the soil for subsequent growing seasons (Kaye and Quemada, 2017). In the global context of regenerative and conservation agriculture, cover crops have been identified as a key component for ensuring long-term sustainability and ecological resilience of agroecosystems (Marandola et al., 2019; Aiyer et al., 2022; Inveninato Carmona et al., 2022). While cover crops can advance sustainability goals in crop production, their improper implementation could lead to a reduction in the yield of the primary crop. A large-scale remote sensing study on the yield reduction introduced by cover cropping showed an average yield loss of 5.5% and 3.5% for corn (Zea mays L.) and soybean (Glycine max L.) fields in the U.S. Corn Belt (Deines et al., 2023). Terminating rye cover crop close to the planting date of cash crop was found to increase the seedling root disease, which led to reduced yield for corn production (Acharya et al., 2022). Planting ruzigrass cover crop was found to reduce the soil phosphorus availability (Almeida et al., 2019). Continuing to advance management practices as “systems” that optimize benefits from cover crops while minimizing cash crop yield penalty is a critical element of their success and extended use (Basche and Roesch-McNally, 2017; Koehler-Cole et al., 2023; Popovici et al., 2023).

The accumulation of above ground biomass (AGB) from cover crops serves as the fundamental driving force behind these potential benefits as well as potential negative impacts on cash crop yield (Ruis et al., 2019; Nichols et al., 2020). Consequently, quantitatively monitoring this process for different cover crops becomes an essential step in assessing their performance. Furthermore, such monitoring could empower farmers with digital tools to support their decision-making for important applications, such as establishing data-driven methodologies to determine optimal termination dates of cover crops, and understanding nutrient release and recycling to cash crops. The biomass accumulation process, however, is complex due to the genotype by environment (GxE) interactions created by differing geographical locations, fluctuating climatic conditions year after year, and variability in farmers’ practices (Koehler-Cole et al., 2023). As such, intensive research is ongoing to determine the optimal types and cultivars of cover crops, the best termination stage, and the suitable termination method for local farmers (Adetunji et al., 2021). Conventionally, cover crop AGB has been measured through destructive sampling of a specific subarea in the plot. This sampling process is time-consuming and labor-intensive, and poses a challenge in experiments constrained by plot size due to the need to balance treatment levels and field size (O'Brien et al., 2022; Zhang et al., 2022; Capri et al., 2023). Nevertheless, understanding the dynamic nature of cover crop biomass accumulation at high temporal resolution could offer insights for researchers seeking to develop or refine predictive models for cover crop research. However, the use of destructive sampling methods often precludes the possibility of continuous quantification of plant materials over the entire experimental period.

Field-based High-Throughput Plant Phenotyping (FHTPP) systems have made significant progress in recent years, owing to the proliferation of low-cost sensors, ground vehicles, unmanned aerial vehicles (UAV), and high-resolution satellite Constellations (Atefi et al., 2020; Zhang et al., 2020; Feng et al., 2021). Some of these systems have facilitated data acquisition at sub-leaf resolution by measuring plants from a close range (Bai et al., 2018; Bai et al., 2019; Yuan et al., 2019). Various Machine Learning (ML) models leverage morphological and spectral traits extracted from FHTPP systems to quantify important agronomic traits, such as canopy height, flowering date, maturity date, and AGB, among others (Zhou and Nguyen, 2021). While substantial advancements have been made in the development of data collection and processing pipelines in FHTPP, there is a paucity of publications documenting its application for quantifying cover crop AGB. Given the growing popularity and potential benefits of cover crops, FHTPP emerges as a promising tool, capable of quantifying the AGB accumulation process in a non-destructive way with notable efficiency. Multispectral imaging has been employed to establish a correlation with the termination efficiency rating for various cover crop species (Kumar et al., 2023). High-resolution satellite images were used to estimate cover crop biomass, achieving the highest correlation coefficient (R) of 0.74 (Kharel et al., 2023). It is worth noting that some crop species, such as winter wheat, can be utilized as both cash and cover crops, hence a relatively larger volume of literature is available for them. Multispectral images, gathered by UAVs, have been widely used to estimate the AGB of winter wheat with the aid of ML models (Wang et al., 2022). Similarly, Vegetation Index–based ML models have been developed and compared for the estimation of AGB of potato (Solanum tuberosum L.) canopy (Liu et al., 2022a; Liu et al., 2022b). Both traditional ML and deep learning models have been explored for AGB estimation, the choice of which depends on the size of the available data (Yue et al., 2018; Dong et al., 2020; Whitmire et al., 2021).

We undertook a two-year experiment involving five cover crop species: canola, rye, triticale, vetch, and wheat. The objectives of this study were: 1) to employ a FHTPP system to collect high-resolution phenotypic datasets for the cover crops, and 2) to build and evaluate ML models to quantify AGB of cover crops. This research aims to fill an important gap in the application of FHTPP in cover crop research, potentially leading to new tools for cover crop breeding and field management.

## 2 Materials and methods

### 2.1 Experimental design and ground truth data collection

**Figure 1:**
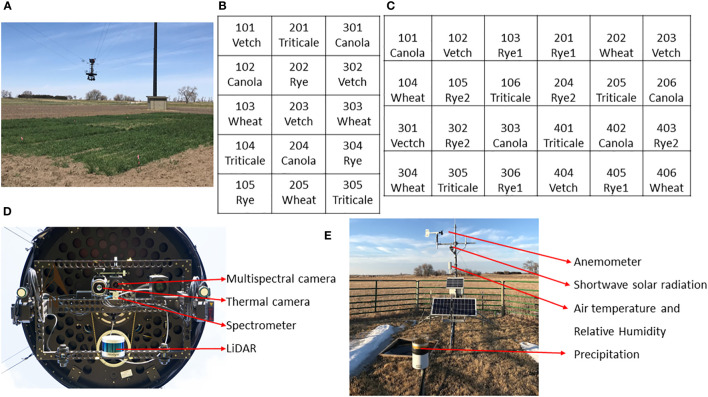
Field condition, experimental layout, and instrumentation of the experiment. **(A)** Field photo of the data collection; **(B, C)** Experimental layout in 2022 and 2023 seasons, respectively; **(D)** Sensors onboard the NU-Spidercam platform; **(E)** Instrumentation of the on-site weather station.

**Table 1** provides details of a two-year cover crop experiment conducted on a research farm near Mead, Nebraska, United States (41°08’44” N, 96°26’20” W, and an elevation of 350m). The study spanned the growing seasons of 2022 and 2023, involving the cultivation of five cover crop species, with no irrigation or nitrogen application. The dominant soil types are Filbert silt loam and Yutan silt clay loam. **Figure 1** illustrates the field conditions where data collection took place, the experimental designs employed, and the cameras/sensors used in the study. In both years, five cover crop species were planted, with each year having 3 and 4 reps, respectively. In 2023, the experiment included two different rye varieties. This gives a total of 15 plots in the first year, and 24 plots in the second year. The dimensions of the plots were 4.6 m by 6.1 m in 2022 and 1.9 m by 6.1 m in 2023. At each plot, a designated area of 0.5 m² was marked using flags. Cover crops and any additional green vegetation within these areas were hand-clipped from the soil surface (**Supplementary Figure S1**). The harvested fresh biomass was oven-dried at 65°C until a constant weight was achieved for dry AGB determination.

**Table 1:** Information for the two-year cover crop field experiment.

Year	Parameter	Information
2022	Cover crop species and cultivars	Vetch: Hairy vetch Canola: Trophy canola Wheat: Winter wheat Triticale: Triticale Rye: Yankee rye
	Planting date	Sep-26-2021
	Biomass sampling dates	Apr-18-2022 Apr-26-2022 May-09-2022
2023	Cover crop species and cultivars	Vetch: Hairy vetch Canola: Trophy canola Wheat: Winter wheat Triticale: Triticale Rye: Elbon and Yankee rye
	Planting date	Sep-27-2022
	Biomass sampling dates	Apr-07-2023 Apr-27-2023 May-08-2023

All crop species survived the winter of the first year, although there was a notable reduction in canola AGB in the spring. In contrast, triticale, vetch, and canola were winterkilled in the second year, attributable to a uniquely dry and cold regional winter. Destructive AGB sampling was carried out three times each year in the spring, resulting in a total of 45 and 72 AGB samples for the respective years (including winterkilled plots with a yield of 0 kg/ha in 2023). These samples include 36 plots with zero AGB due to winter kill in the spring of 2023. The plot-scale HTPP data, including multispectral images, LiDAR (Light Detection and Ranging) point clouds, thermal images, and spectral reflectance in the visible and near-infrared range, were collected using a cable-suspended field phenotyping system. Additionally, an on-site weather station recorded standard weather data at a 1-minute interval, ensuring an accurate integration of the phenotypic and environmental data.

### 2.2 Data processing before machine learning

**Figure 2:**
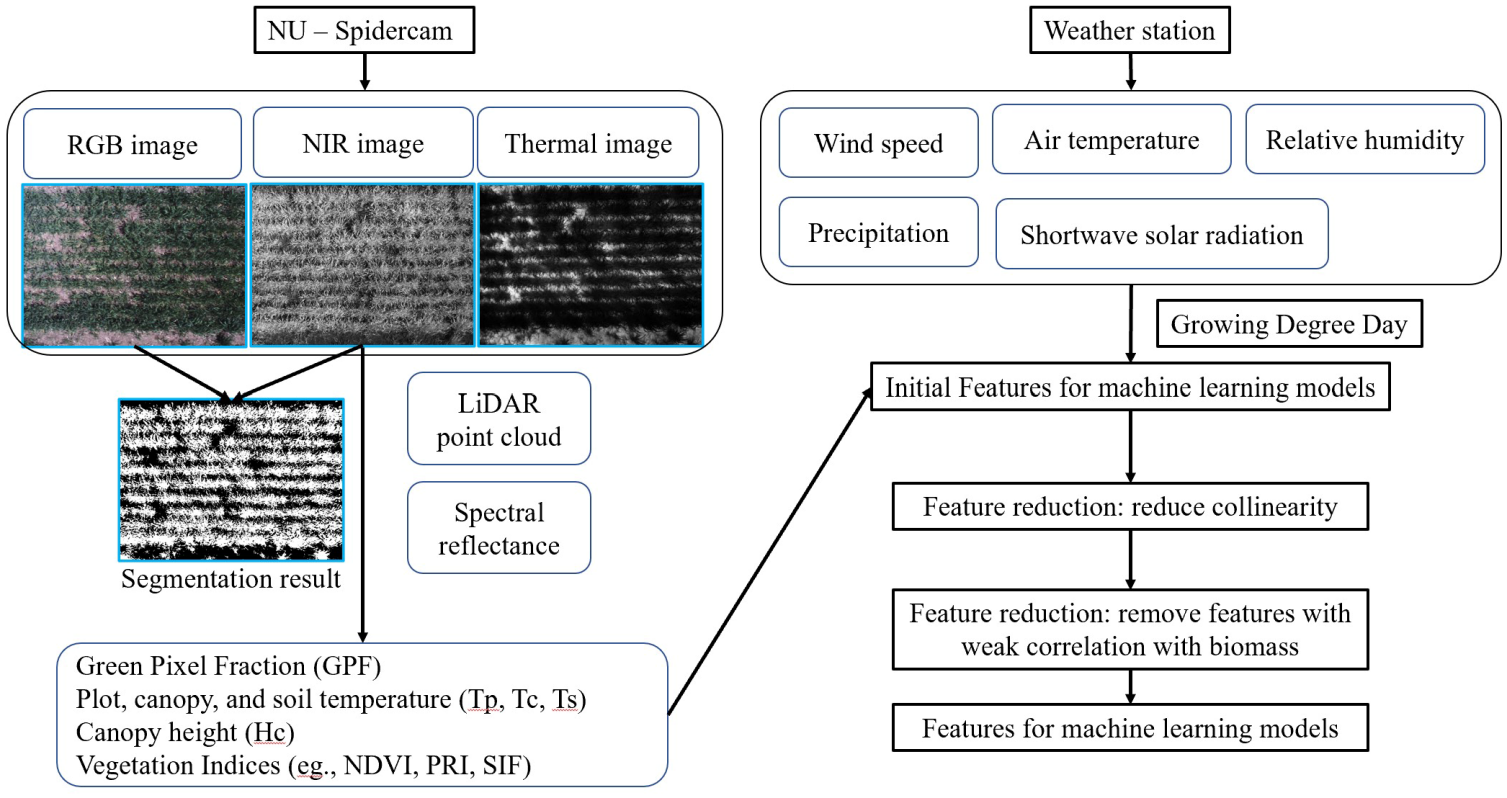
Data processing pipeline for phenotypic and environmental features. The left panel visualizes the data processing of NU-Spidercam data, including raw images and a list of phenotypic parameters; the right panel lists the raw environmental data collected from the on-site weather station and the feature reduction process before the development of machine learning models.

**Figure 2** outlines the data preprocessing pipeline employed for feature extraction in the context of ML modeling. Phenotypic data, anticipated to correlate with plant AGB, were derived from raw sensor data following a previously developed data processing protocol (Bai et al., 2019). Environmental data, synchronized with the phenotypic data via timestamps, were also incorporated into the analysis. The phenotypic and environmental features are listed in **Figure 2**. A set of raw images were also presented alongside their corresponding segmentation results. For each dataset, nadir-captured multispectral and thermal images were obtained using the sensing platform. Green vegetation pixels were identified as foreground through image registration, histogram equalization, and thresholding. Green Pixel Fraction (GPF), plot average temperature (Tp), canopy average temperature (Tc), and soil average temperature (Ts) were computed based on the segmentation outcomes. GPF demonstrated a strong correlation with Normalized Difference Vegetation Index (NDVI) throughout the growing season, suggesting its potential as a robust estimator for quantifying canopy growth before canopy closure (Bai et al., 2016). Tp contributed to the estimation of the Leaf Area Index and was used to detect crop water stress (Irmak et al., 2000; Wang et al., 2021; Cheng et al., 2023). Additionally, Growing Degree Day (GDD), a widely adopted feature in crop growth models, was calculated as an additional environmental feature using weather data from the first day of the year. Two base temperatures were applied for the GDD calculation (Winter wheat: 0°C (Slafer and Savin, 1991); Canola: 5°C (Lawson et al., 2006); Rye: 0°C (Szuleta et al., 2022); Vetch: 5°C (Lawson et al., 2012); Triticale: 0°C (Schwarte et al., 2006). Cover crops sharing the same base temperatures had identical GDD values (**Supplementary Equation S1**). A comprehensive list of equations used for calculating Vegetation Indices (VIs) is provided in the **Supplementary File** (**Supplementary Table S1**), sourced from prior publications (Rouse et al., 1974; Rondeaux et al., 1996; Gamon et al., 1997; Barnes et al., 2000; Suárez et al., 2008; Meroni et al., 2009; Badgley et al., 2017).

### 2.3 Machine learning models for aboveground biomass estimation

Feature reduction was carried out to mitigate strong multicollinearity among features, as well as to exclude features that showed a weak correlation with AGB (|R| < 0.45). Subsequently, one-hot encoding was applied to convert the crop species into a numerical feature. Model performance was investigated using all features first and using different categories of features. The input features for model training were categorized into phenotypic and environmental features. Phenotypic features can quantify the variance among individual plots due to growth heterogeneity caused by differences in soil, water, and nutrients. Phenotypic features were further grouped into morphological (GPF and Canopy Height (Hc)), spectral (VIs), and thermal (Tp) groups, which were captured using different onboard instruments (**Figures 1**, **2**).

Given the small data size, we used non-deep learning models to avoid the risk of overfitting to a certain degree. These models include Random Forests Regression (RFR), Support Vector Regression (SVR), Partial Least Squares Regression (PLSR), and Artificial Neural Network (ANN). RFR is an ensemble learning method that constructs multiple decision trees during training and outputs the mean prediction of the individual trees for AGB modeling. This method not only offers robustness against overfitting but also provides an inherent feature importance evaluation, enabling an understanding of which predictors are most influential in the modeling process. SVR is a regression adaptation of support vector machines, which operates by identifying an optimal hyperplane that functions as a decision boundary. It strives to ensure that most data points are within a certain margin of this hyperplane, maximizing the margin while limiting the regression errors. This method is effective in providing robust predictions, particularly for datasets with noisy observations. PLSR is a modeling method specializing in handling highly correlated input features. It works by transforming the original input features into a smaller set of uncorrelated components, capturing the most variance in the dataset. These components are formed from linear combinations of the original variables and are used in building the model to efficiently deal with multicollinearity and reducing dimensionality. ANNs are powerful computational models that excel at capturing complex non-linear relationships. They function by adjusting weights and biases during training to minimize a specific loss function and improve the prediction accuracy. This process involves multiple training cycles of forward and backward propagation. By iteratively refining these parameters, ANNs learn high-level patterns and correlations that are often missed by more traditional statistical methods. During the development of the ANN model, only two hidden layers were used, both incorporating potential L2 regularizations to reduce overfitting. The primary parameters explored during hyperparameter tuning are detailed in **Supplementary Table S2**. The dataset was randomly split into training (80%) and test (20%) sets. Data standardization was carried out for each feature independently. During model training, ten-fold cross-validation was implemented across all models. The optimized models with the best hyperparameters were selected based on the highest R^2^ observed during cross validation. The chosen optimal model was then implemented for predicting AGB in the test set, and the predictive performance was assessed using R^2^, Root Mean Squared Error (RMSE), and Normalized RMSE (NRMSE). Scikit-learn, a Python library, was used for training and evaluating ML models. Mean decrease impurity, coefficient magnitudes, and PLS loadings were employed to evaluate feature importance in RFR, SVR, and PLSR models, respectively.

## 3 Results and discussion

### 3.1 Aboveground biomass and data pre-processing

**Figure 3:**
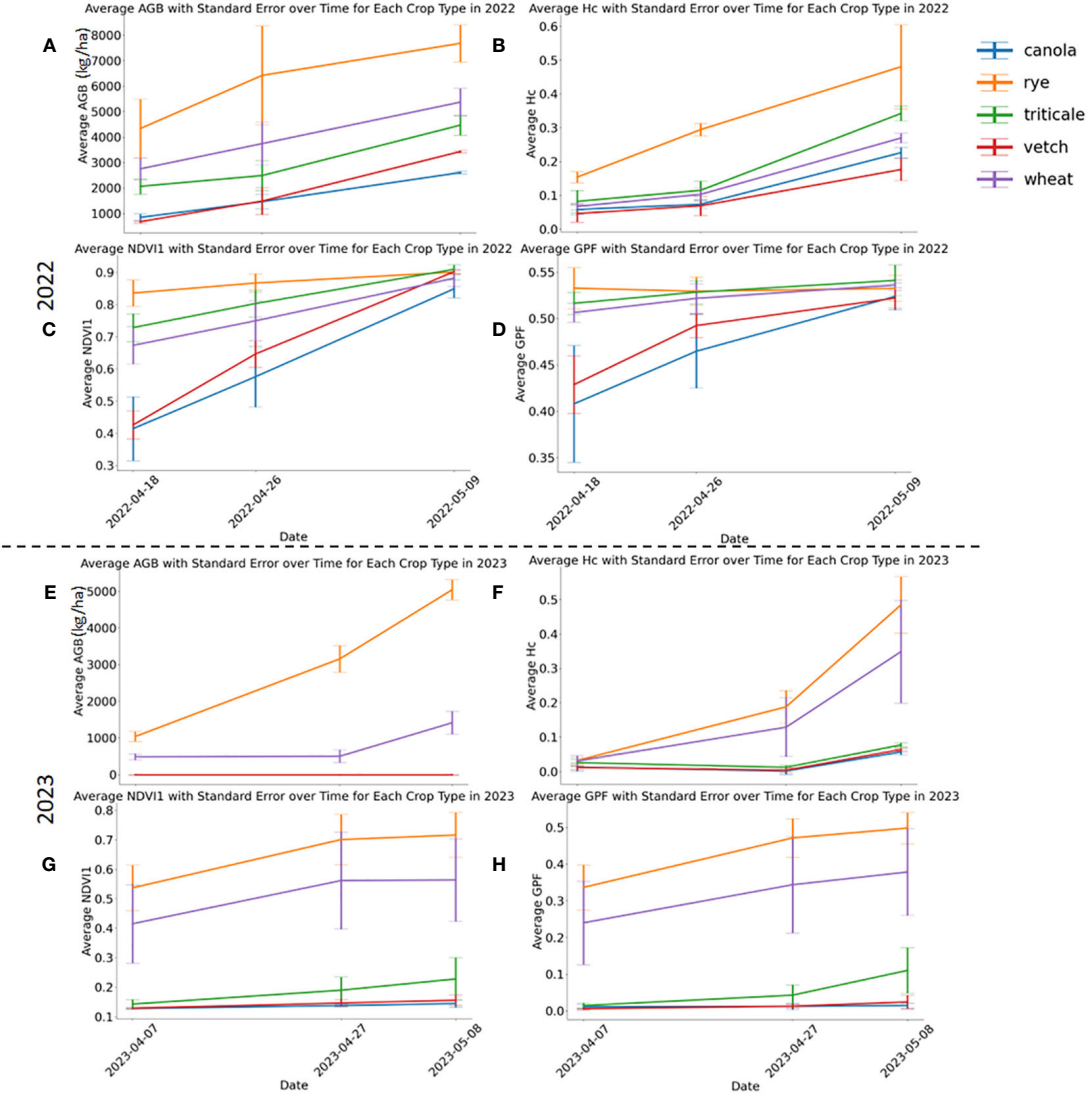
Aboveground biomass and selected phenotypic parameters in 2022 **(A–D)** and 2023 **(E–H)**. Three phenotypic parameters include Hc, NDVI1, and GPF. Standard errors are marked with vertical bars.

Descriptive statistics of AGB for each crop type in the whole, training, and testing data sets are illustrated in the **Supplementary Document** (**Supplementary Figure S2**). **Figure 3** shows the temporal variations of AGB and selected sensor-based phenotypic features (Hc, NDVI1, and GPF) on the three sampling dates in both years. All parameters exhibited a consistent increase over time, aligning with the observed AGB growth, thus suggesting their potential as features to estimate cover crop AGB. In both years, rye consistently displayed the highest biomass, surpassing other cover crops significantly (**Figures 3A, E**). Notably, rye stands out as the most widely grown cover crop species in the U.S. Midwest (Nichols et al., 2020). The capacity to produce exceptional AGB under the local climate has prompted the wide adoption of rye cover crop (Ruis et al., 2019; Koehler-Cole et al., 2020). In addition, rye’s capability to significantly suppress weed growth leads to reduced management costs (Oliveira et al., 2019; Rosa et al., 2021). A field study found that a minimum AGB of 5000 kg/ha was required to achieve a 75% reduction in weed biomass (Nichols et al., 2020). Based on the biomass data obtained in this study, it is evident that rye was the only species consistently achieving this threshold in both years, typically by the end of April or the beginning of May, given a planting date at the end of September. In 2022, wheat and triticale outperformed vetch and canola in terms of biomass production. A substantial portion of the canola perished in the spring of 2022, resulting in the lowest biomass yield.

Rye consistently demonstrated higher Hc values than other cover crops, and all species exhibited increasing Hc values throughout the experimental period (**Figure 3B**). At the first and second sampling dates of 2022, rye had the highest NDVI1 and GPF values (**Figures 3C, D**). Discrepancies in VIs among the cover crop species diminished by the second date, ultimately reaching the same level by the third sampling date in 2022. By the final sampling day of 2022, NDVI1 and GPF for different crop species were not significantly different. However, these two parameters for vetch and canola plots experienced a faster increase than those of others during the investigated period. In 2023, triticale, vetch, and canola experienced winterkill, potentially attributable to diminished snow cover during the coldest periods. A few triticale plants survived in 2023 (**Supplementary Figure S3**), resulting in slightly higher NDVI1 and GFP than vetch and canola, which were essentially bare soil (**Figures 3G, H**). This underscores a potential risk associated with growing less winter-hardy crops, such as legumes and brassicas, in the study region. Between rye and wheat, all assessed phenotypic parameters showed higher values for rye than wheat in 2023 (**Figures 3F–H**). The development of additional features can be found in the **Supplementary Document** (**Supplementary Figures S4**, **S5**).

### 3.2 Correlation analysis

**Figure 4:**
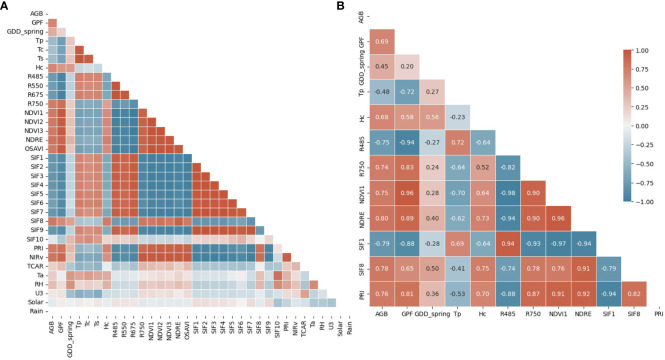
Correlation matrix of all **(A)** and reduced features **(B)**, including morphological (GPF and Hc), spectral (VIs), thermal (Tp, Tc, and Ts), and environmental features (GDD_spring, Ta, RH, U3, Solar, and Rain). The correlation coefficient is shown in the correlation matrix of reduced features.

The correlation matrix of all features and AGB before the feature reduction for all crop species is shown in **Figure 4A**. We categorized these features into four groups for further analysis: morphological (GPF and Hc), spectral (all VIs), thermal (Tp, Tc, and Ts), and environmental (all features from the on-site weather station). The first feature reduction aimed to eliminate features with strong collinearity while retaining at least one feature from each category. Tp, the average temperature of all canopy and vegetation pixels, was retained as the representative thermal feature for Tc and Ts due to their strong positive correlations. Further investigation revealed a notable temperature difference in mid April between Tc and Ts, as opposed to early May (**Supplementary Figure S6**). However, Tp consistently showed very strong correlations with Tc and Ts. Another rationale for retaining Tp as the thermal feature, rather than Tc or Ts, was its relatively easier measurability without a need to distinguish between canopy and soil pixels. A previous study showed that thermal parameters improve ML-based prediction of the Leaf Area Index for maize (*Zea mays* L.) (Wang et al., 2021). Therefore, we expected that integrating Tp would improve the model’s performance in estimating AGB. R485 (Blue) and R675 (Red) represent the strong absorption bands by the crop canopy, while R550 (Green) denotes the most reflected band in the visible range. Due to their strong positive correlations, reflectance in the blue wavelength (R485) was kept to represent spectral reflectance in the visible wavelength range. All NDVIs, Normalized Difference Red Edge (NDRE), and Optimized Soil Adjusted Vegetation Index (OSAVI) exhibited strong positive correlations due to the similar wavelengths used in their calculations. Thus, NDVI1 and NDRE were retained for NDVI-related VIs. NDRE was also kept due to its lesser susceptibility to saturation, as evidenced by its behavior at the final sampling date (**Supplementary Figures S4**, **S5**). Among the ten Solar-induced Fluorescence (SIF) features, only SIF1, SIF8, and SIF10 were kept, as the remaining SIFs had strong positive correlations with SIF1. These strong correlations could be attributed to similarities in their calculation equations. Notably, SIF8 uses reflectance values from two NIR bands, which is distinct from the equations of other SIFs. While most SIF features leverage reflectance near an oxygen absorption band (O2-B) around 687 nm, SIF10 utilizes reflectance near another oxygen absorption band (O2-A) around 760 nm. The results of the first feature reduction are available in the **Supplementary Document** (**Supplementary Figure S7**).

The second feature reduction involved the removal of features with a weak correlation to AGB (|R| < 0.45). In this step, all environmental features were filtered out except for GDD from the beginning of the year (GDD_spring). This indicates that instantaneous environmental features exerted a weaker influence on AGB than phenotypic parameters in this study. The only accumulative parameter, GDD_spring, demonstrated relatively stronger predictive power for AGB (R = 0.45). SIF10 and Transformed Chlorophyll Absorption in Reflectance (TCAR) were also excluded due to their weak correlation with AGB. When considering crop species as a single feature, the feature reduction process resulted in the selection of 12 features for ML model training. **Figure 4B** illustrates the correlation matrix of the final features, including an environmental feature (GDD_spring), two morphological features (GPF and Hc), seven spectral features (seven VIs), and a thermal feature (Tp). These features served as inputs for ML models, although strong correlations persisted among some of them.

**Figure 5:**
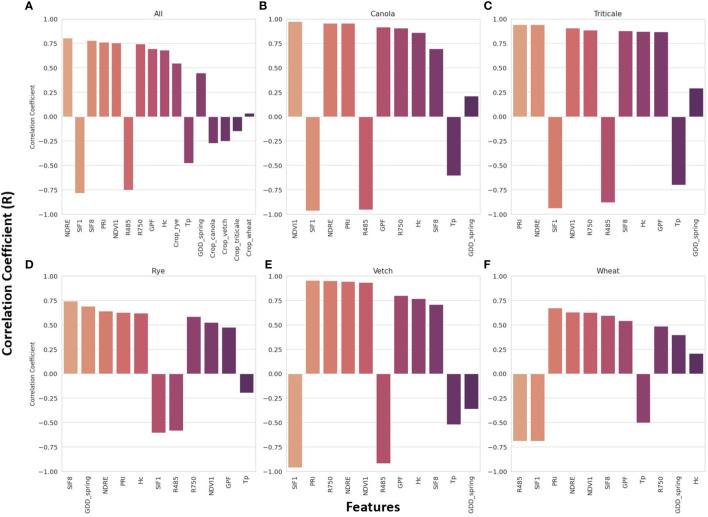
Pearson’s correlation coefficient (R) between all features extracted from the images and sensor data and the aboveground biomass for all crop species **(A)** and each specific crop species **(B–F)**.

**Figure 5** presents the sorted R values between feature inputs and AGB for all crop species (**Figure 5A**), as well as for each specific crop species (**Figures 5B–F**). In general, the top five features with stronger correlations were VIs calculated from spectral reflectance. Specifically, NDRE (R = 0.8), SIF1 (R = -0.79), SIF8 (R = 0.78), PRI (R = 0.76), and NDVI1 (R = 0.75) exhibited the highest correlations with AGB when considering all crop species. For individual crop species, additional features were included in the top five features, including R485, R750, GDD_spring, and Hc. A weighted scoring system was used to rank the overall top five predictors, assigning decreasing scores (from 5 to 1) based on the features’ ranks (1 to 5) for each crop species. The results reaffirmed that VIs exhibit relatively stronger linear correlations with AGB, with a slightly varied ranking compared to **Figure 5A**, where the top five predictors were SIF1, PRI, NDRE, NDVI1, and R485.

### 3.3 Machine learning model performance

**Figure 6:**
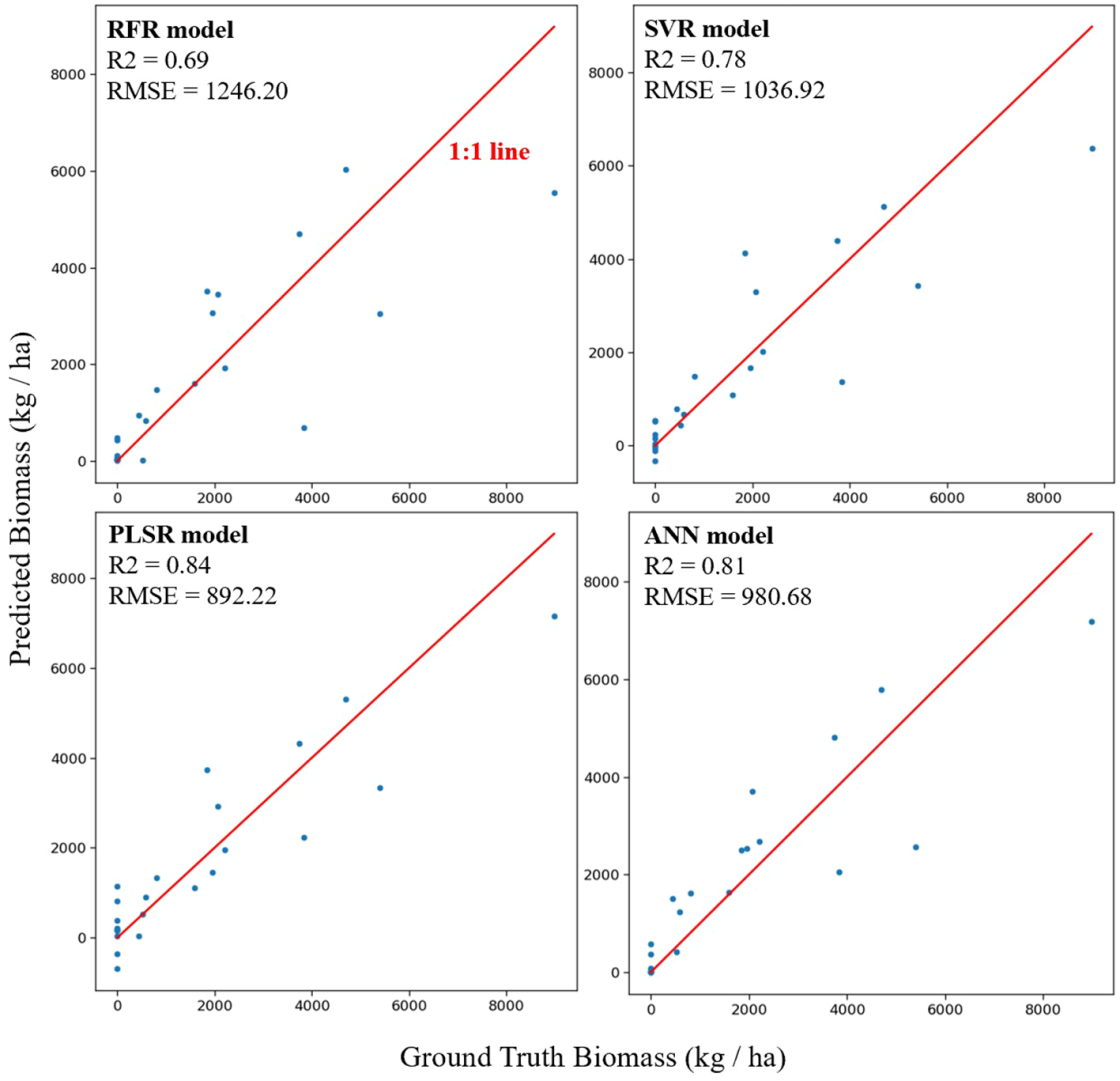
Scatter plots of predicted vs. actual aboveground biomass using all features extracted from the cameras and sensors by four machine learning techniques. Model names and performance metrics (R^2^ and RMSE) are shown in the corresponding subplots.

**Figure 6** illustrates the model performance using all feature inputs across different models. The optimal hyperparameters for each model are listed in **Supplementary Table S3**. R^2^ and RMSE values are also presented in the corresponding subfigures. The PLSR model (R^2^ = 0.84, RMSE = 892 kg/ha, NRMSE = 9%) exhibited the best performance when compared with models that could better capture non-linear interrelationships between the features and AGB. The RFR model (R^2^ = 0.69, RMSE = 1246 kg/ha, NRMSE = 12%) showed the poorest performance, while SVR (R^2^ = 0.78, RMSE = 1037 kg/ha, NRMSE = 10%) and ANN (R^2^ = 0.81, RMSE = 981 kg/ha, NRMSE = 10%) showed comparable performance with each other. These results indicate that AGB can be estimated using linear ML models with good performance. The correlation analysis above also confirmed strong linear correlations between features and AGB with feature collinearity at a certain degree (**Figures 4**, **5**), indicating that PLSR could be the most suitable model for this scenario. Previous work concluded that PLSR showed promising performance in AGB estimation for winter wheat at different growth stages (Wang et al., 2022). Thus, our work further establishes the promising performance of PLSR in AGB estimation for multiple cover crop species. Another study explored AGB estimation for multiple oats (*Avena sativa* L.) cultivars at different locations using RFR, SVR, PLSR, and ANN models. The results showed that no single model performed best across all locations, and PLSR outperformed other models at certain experimental locations (Sharma et al., 2022). Furthermore, a systematic investigation of model performance involving eight ML models concluded that PLSR was among the best models for ABG estimation in winter wheat (Yue et al., 2018). These studies reconfirmed that linear-based models such as PLSR can outperform more complex, non-linear models in AGB estimation. To the best of our knowledge, no prior results are available for comparison with our model performance across multiple cover crop species. Therefore, we exclusively compared the model performance for available crop species, such as winter wheat. Comparable model performance was observed when using drone-based multispectral camera systems and PLSR to estimate winter wheat AGB [R^2^ = 0.75, (Wang et al., 2021)]. A higher model performance was achieved when using VIs from a close-range field spectrometer and PLSR for the same purpose [R^2^ = 0.89, (Yue et al., 2018)]. Additionally, comparable performance was achieved for potato AGB estimation using RFR at the tuber-growth stage [R^2^ = 0.68, (Liu et al., 2022a)].

**Figure 7:**
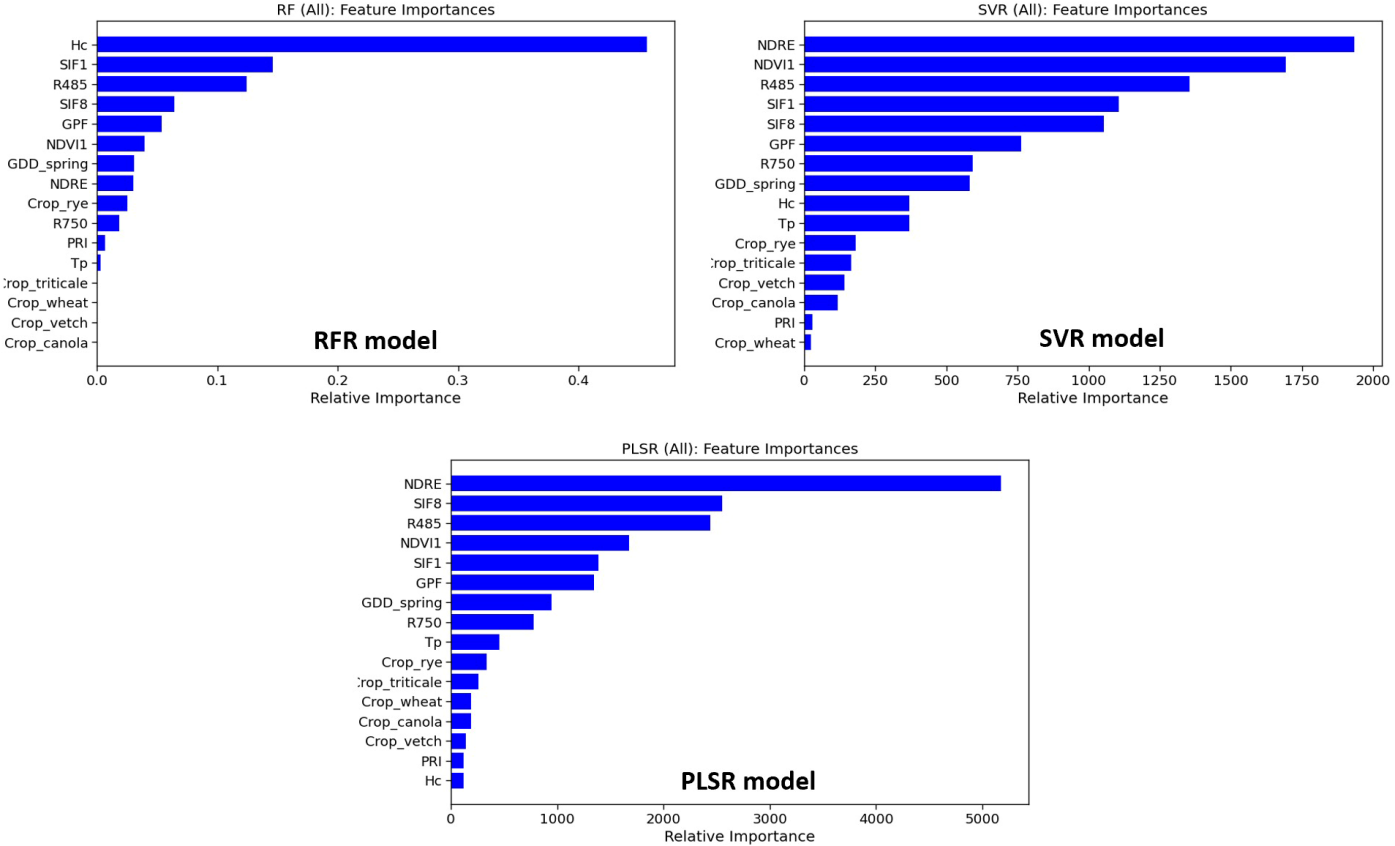
Feature importance for three investigated models, including RFR, SVR, and PLSR.

**Figure 7** shows the feature importance of RFR, SVR, and PLSR models. Morphological features (Hc and GPF) ranked among the top five important features of the RFR model. In SVR, Hc and GPF ranked 9^th^ and 6^th^, while in PLSR, they ranked 16^th^ and 6^th^. SVR and PLSR shared the top five features from VIs with different ranking orders, including NDRE, NDVI1, R485, SIF1, and SIF8. Among these five VIs, SIF1, R485, and SIF8 were also among the top five important features of RFR. The environmental feature, GDD_spring, ranked 7^th^, 8^th^, and 7^th^ for RFR, SVR, and PLSR models, respectively, indicating its significant contribution to model performance. The thermal feature, Tp, ranked 12^th^, 10^th^, and 9^th^ in the three models, respectively, suggesting a slightly lesser contribution to model performance in this study. Generally, the crop species feature held lower importance than other features, with average rankings at 13^th^, 13^th^, and 12^th^ for the three models, respectively.

**Table 2** shows the model performance using different feature categories. Firstly, only morphological features were used in the model development. Except for the ANN model (R^2^ = 0.80, RMSE = 986 kg/ha, NRMSE = 9.81%), other models yielded the lowest R^2^ (0.34 - 0.50) with the highest RMSE and NRMSE (1573 - 1802 kg/ha and 15.64% - 17.92%) compared to other conditions. ANN appears to be the only method capable of estimating AGB for multiple cover crop species with GPF and Hc alone. When using only spectral parameters as feature inputs, ANN still demonstrated the highest R^2^ (0.77), although with a slightly reduced margin compared to other methods (RFR: 0.66; SVR: 0.62; PLSR: 0.68). The relatively strong performance of using spectral features alone (R^2^ ≥ 0.62) confirms the application of aerial sensing platforms with a single multispectral camera (e.g., drones and high-resolution satellite imagery) for cover crop AGB estimation. The difference in model performance between the models utilizing spectral features and those using both morphological and spectral features was small (ΔR^2^ = ± 0.2). This result indicates that GPF and Hc did not improve the model performance when spectral features were already utilized. A possible explanation is that the spectral features already embedded the useful information of GPF and Hc. Upon excluding the crop species feature, model performance declined except for the ANN model. The feature importance of crop species for RFR, SVR, and PLSR models also explains to a certain degree why adding this feature improves the performance of the overall model for all five crop species. The impact of the thermal feature, Tp, on model performance was also investigated by estimating model performance without Tp. The results indicated that Tp contributed the least to the improvement of R^2^ and RMSE for RFR, SVR, and PLSR models. Notably, including Tp led to a higher RMSE for ANN. Therefore, the instantaneous thermal features (e.g., Tp) may offer limited improvement in this application. Overall, we concluded that accurate AGB estimation for multiple cover crop species can be achieved using drone systems or high-resolution satellite constellations, which provide spectral and morphological features at the plot level. Also, more accurate prediction models can be generated from drone systems due to their higher spatial resolution of images, while satellite systems offer benefits in terms of higher throughput and coverage (Sankaran et al., 2015; Zhang et al., 2020; Kharel et al., 2023). Furthermore, accurate canopy height and thermal features could improve the model performance to a certain extent.

**Table 2:** Comparison of model performance for cover crop aboveground biomass estimation by categorizing model inputs into morphological, spectral, and thermal groups. The units of RMSE and NRMSE are kg/ha and %, respectively.

Model	Metric	Feature Selection					
		Morphological (GPF, Hc)	Spectral	Morphological And Spectral	All but crop species	All but thermal (Tp)	All
RFR	R^2^	0.34	0.66	0.67	0.65	0.68	0.69
	RMSE	1802	1287	1279	13201	1253	1246
	NRMSE	17.92	12.80	12.72	131.28	12.46	12.39
SVR	R^2^	0.50	0.62	0.63	0.67	0.75	0.78
	RMSE	1573	1365	1355	1273	1109	1037
	NRMSE	15.64	13.57	13.48	12.66	11.03	10.31
PLSR	R^2^	0.44	0.68	0.67	0.72	0.82	0.84
	RMSE	1664	1259	1281	1176	947	892
	NRMSE	16.55	12.52	12.74	11.69	9.42	8.87
ANN	R^2^	0.80	0.77	0.79	0.84	0.81	0.81
	RMSE	986	1063	1008.05	895	958	981
	NRMSE	9.81	10.57	10.02	8.90	9.53	9.76

### 3.4 Model limitation

Based on **Table 2**, the PLSR model built on all features yields the highest R^2^ value of 0.84, with an RMSE of 892 kg/ha. Given this RMSE value, the current model’s accuracy may require further improvement to assist producers in predicting aboveground performance from advanced technology. Additionally, understanding the quantitative influence of the AGB of different cultivars of promising crops (e.g., various rye cultivars) on potential benefits is necessary. This effort will help establish clear target RMSE ranges of the models. The models developed in this study were trained and tested on multiple cover crop types using data from two years. However, the study was limited to a single site in Nebraska with specific soil and weather conditions. As the models are purely data-driven, we acknowledge that their performance may not be robust when predicting cover crop AGB in environments beyond the investigated ranges, even within the U.S. Midwest and Great Plains. Further validation of the model’s robustness and transferability is required by testing with truly independent data sets. For instance, a multi-location experiment across Nebraska, from west to east, would provide a much broader range of growing environments for candidate cover crops, aiding in testing and model improvement.

## 4 Conclusion

High-throughput plant phenotyping offers a non-destructive and efficient approach for estimating plant AGB. This study encompassed a two-year field experiment aiming at developing ML models for AGB estimation of five cover crop species - rye, vetch, canola, winter wheat, and triticale - from phenotypic and environmental data in the U.S. Midwest region. The raw dataset included multispectral imagery, LiDAR point clouds, spectral reflectance, thermal images, and environmental data, complemented by the AGB data obtained via destructive sampling. Consistent with prior research in similar climates, rye outperformed other species in terms of AGB accumulation in both years. Leveraging morphological, spectral, environmental, crop species, and thermal features extracted from the raw dataset, we employed four ML techniques - Random Forest Regression (RFR), Support Vector Regression (SVR), Partial Least Squares Regression (PLSR), and Artificial Neural Network (ANN) - to predict cover crop AGB. PLSR emerged as the best approach (R^2^ = 0.84, RMSE = 892 kg/ha, NRMSE = 8.87%) when utilizing all feature inputs. Thus, this study highlights linear models, like PLSR, are on par with non-linear models in capturing the fundamental relationship between features and AGB for cover crop research, especially when dealing with feature collinearity. All feature categories contributed to the performance of RFR, SVR, and PLSR models, while spectral features alone exhibited the strongest performance for RFR, SVR, and PLSR models. Morphological features alone yielded satisfactory results when trained with the ANN model. Instantaneous thermal features made a marginal contribution to the model performance in this study. Despite achieving high testing accuracy in this study, we suggest that further training and validation of the models, using larger datasets and various data splitting techniques, could enhance model robustness. Consistent with challenges identified in previous research on AGB estimation for various crops, the scarcity of ground truth data continues to be a significant obstacle in developing more accurate and robust models, owing to the inherent constraints of field experiments and limited resources. Collecting more biomass data under different soil and climate conditions could further improve the model performance. Continuous development of more universal and robust image processing algorithms for vegetation segmentation, especially in later growing stages with dense vegetation canopy, could improve GPF quantification. Convolutional neural networks could be leveraged to replace manual feature extraction if a much larger biomass data set is available.

## Data availability statement

The raw data supporting the conclusions of this article will be made available by the authors.

## Author contributions

GB: Data curation, Formal analysis, Methodology, Project administration, Software, Validation, Visualization, Writing – original draft, Writing – review & editing. KK-C: Conceptualization, Data curation, Formal analysis, Methodology, Resources, Writing – review & editing. DS: Conceptualization, Data curation, Formal analysis, Methodology, Writing – review & editing. VT: Visualization, Writing – review & editing. AB: Conceptualization, Funding acquisition, Investigation, Methodology, Project administration, Writing – review & editing. YG: Conceptualization, Formal analysis, Funding acquisition, Investigation, Methodology, Project administration, Writing – review & editing.
