# Supplementary material for: Enhancing estimation of cover crop biomass using field-based high-throughput phenotyping and machine learning models
Source: Front Plant Sci. 2024 Jan 8;14:1277672. doi: 10.3389/fpls.2023.1277672 (PMC10800384; doi:10.3389/fpls.2023.1277672)
Supplement: Supplementary file 1 [file DataSheet_1.docx]

Supplementary File


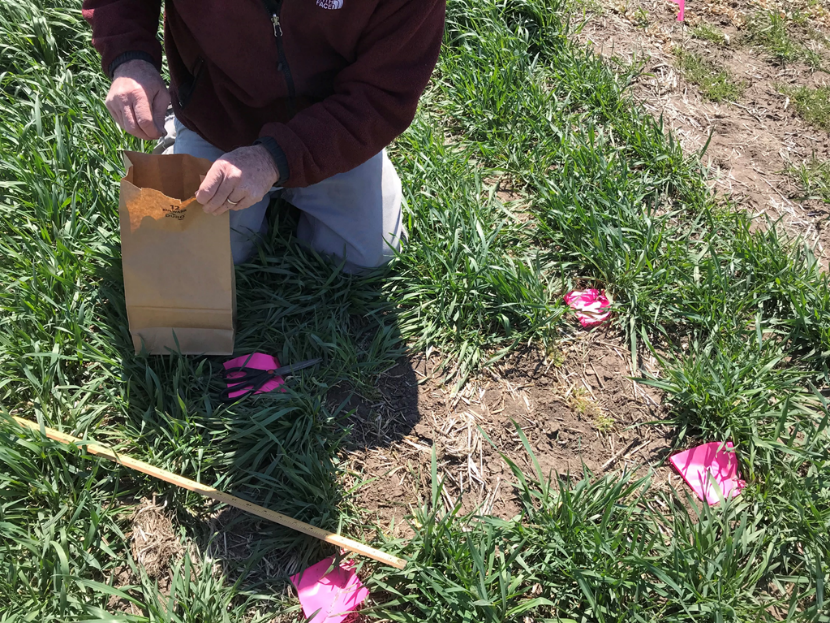


Figure S1. A field photo of destructive biomass sampling


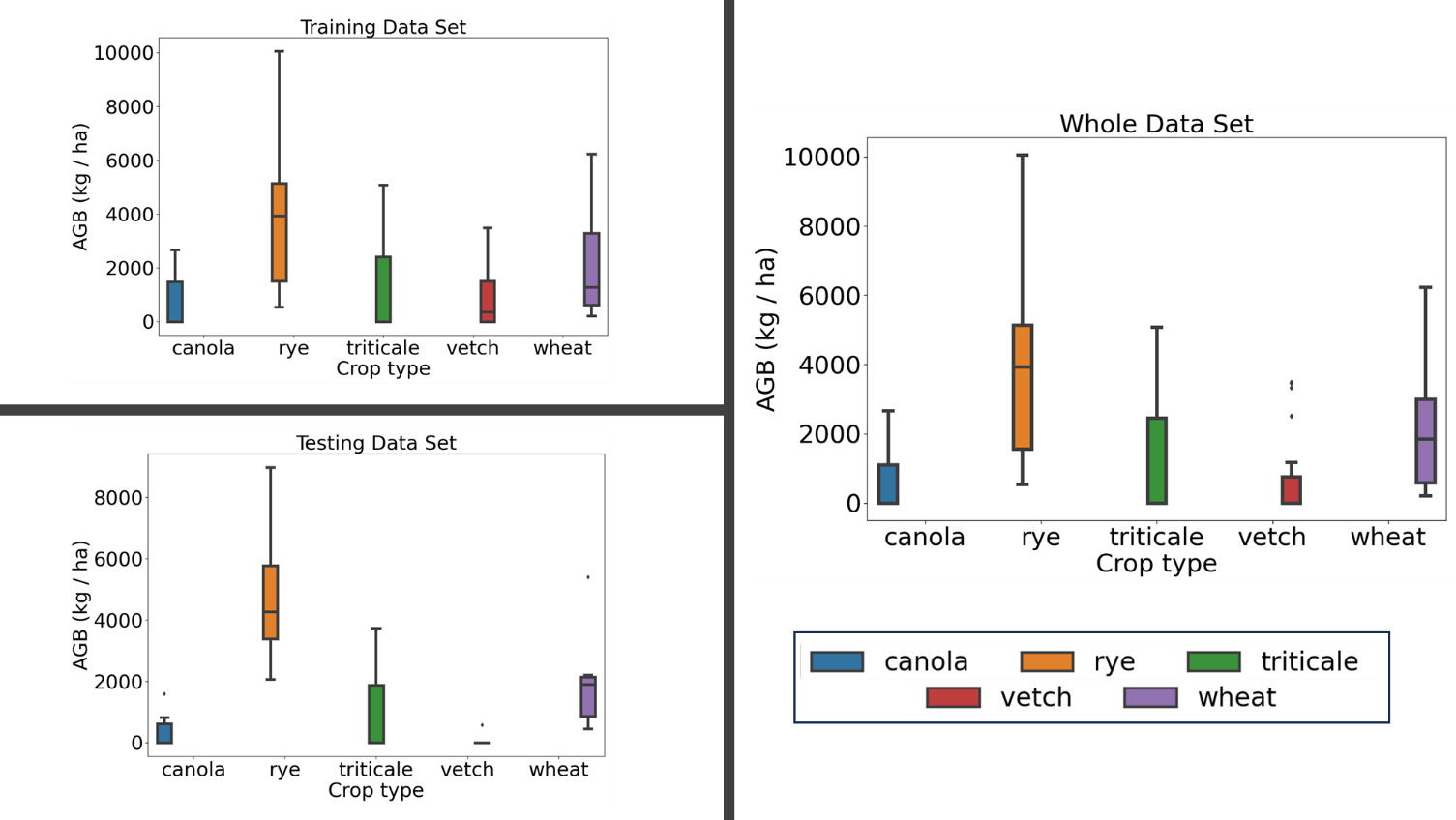


Figure S2. Boxplots of AGB for each crop type in whole, training, and testing data sets.


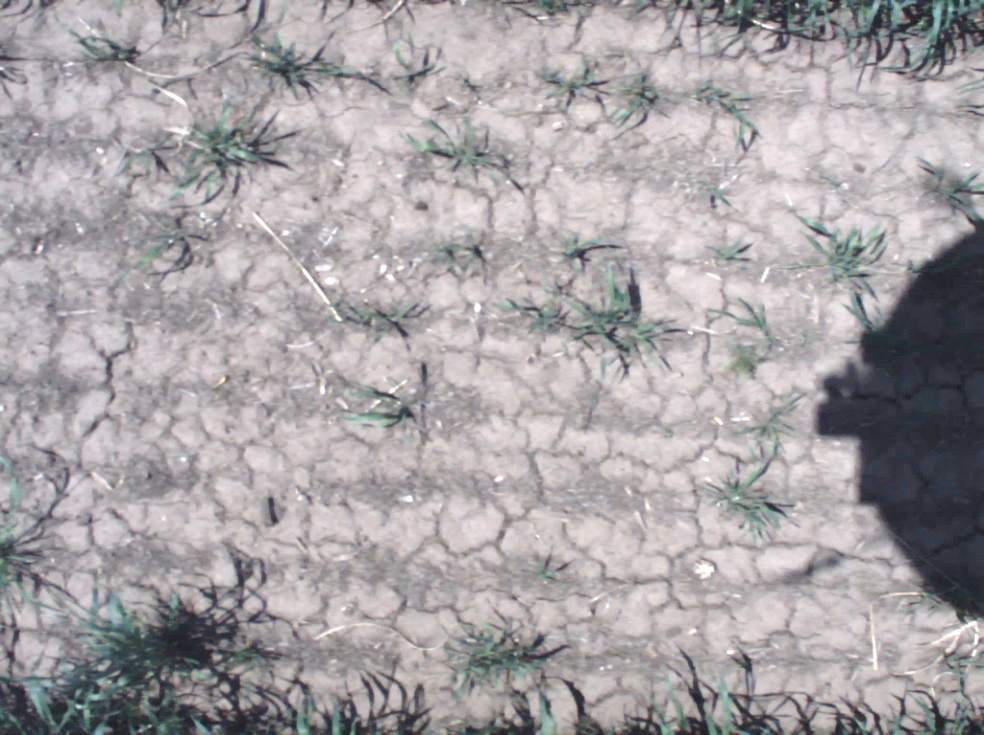


Figure S3. Triticale plots in 2023 with a few plants survived. No biomass sampling was conducted due to the unevenly distributed plants in the plot. Zero biomass was used for these plots.


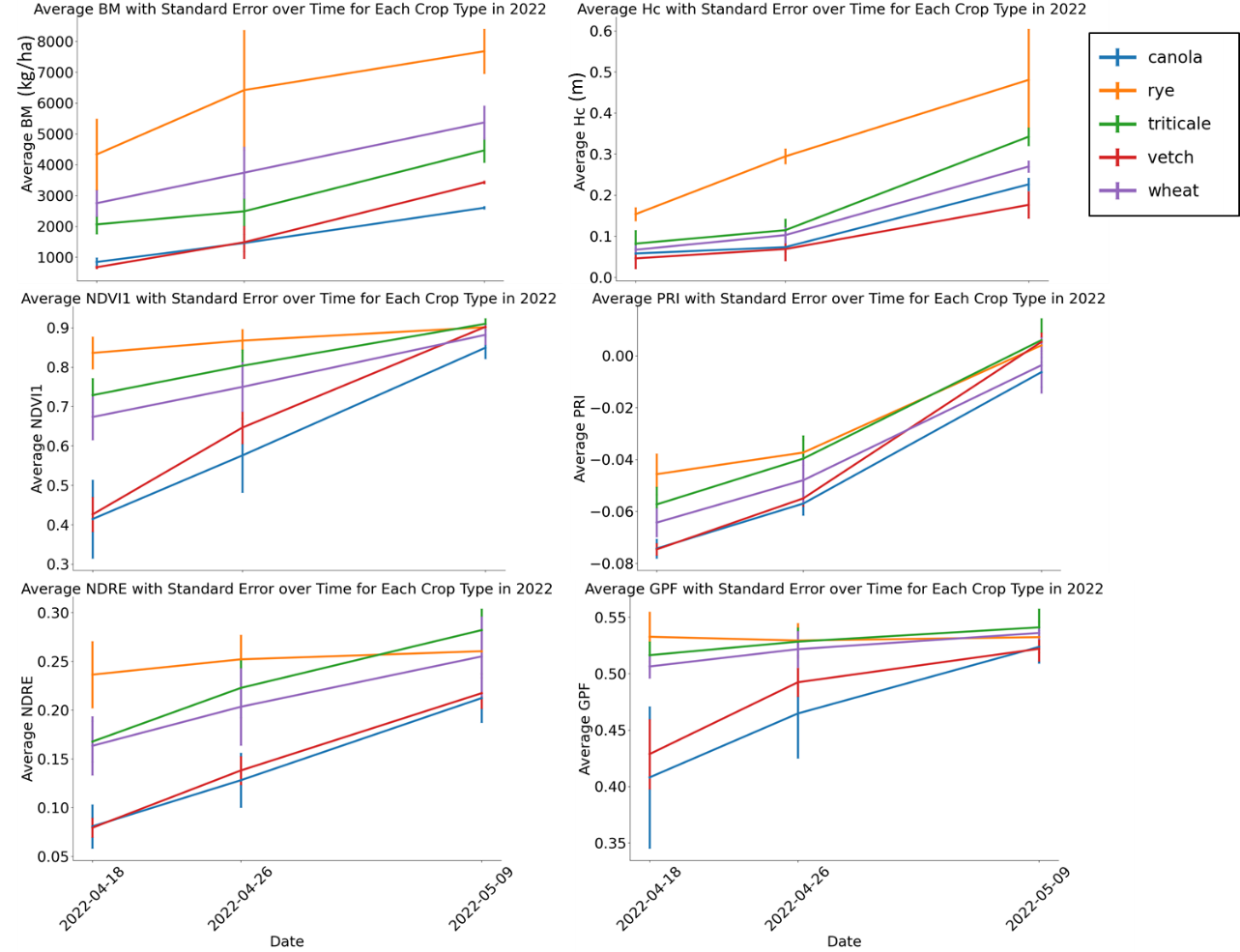


Figure S4 Biomass and selected phenotypic parameters in 2022


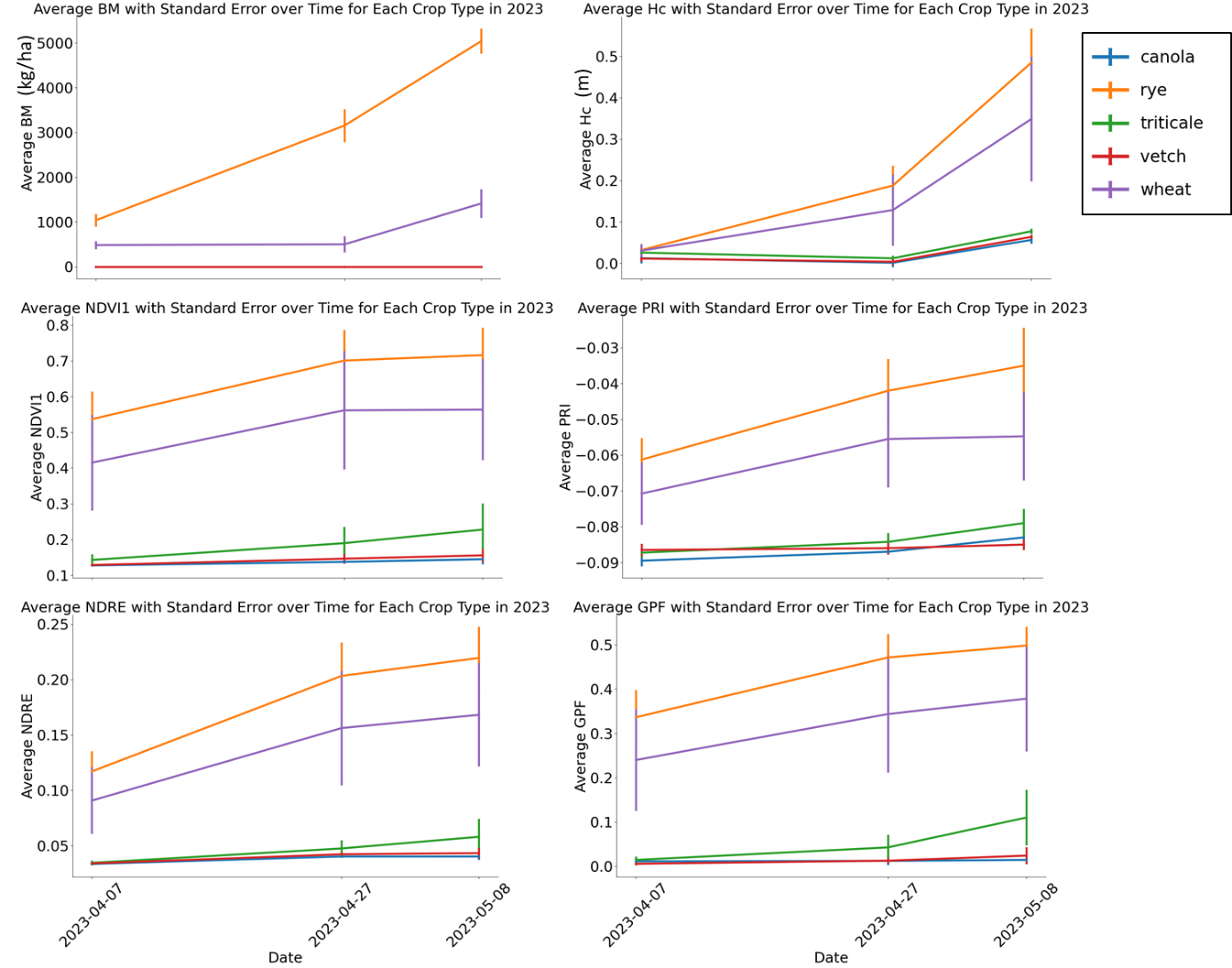


Figure S5 Biomass and selected phenotypic parameters in 2023


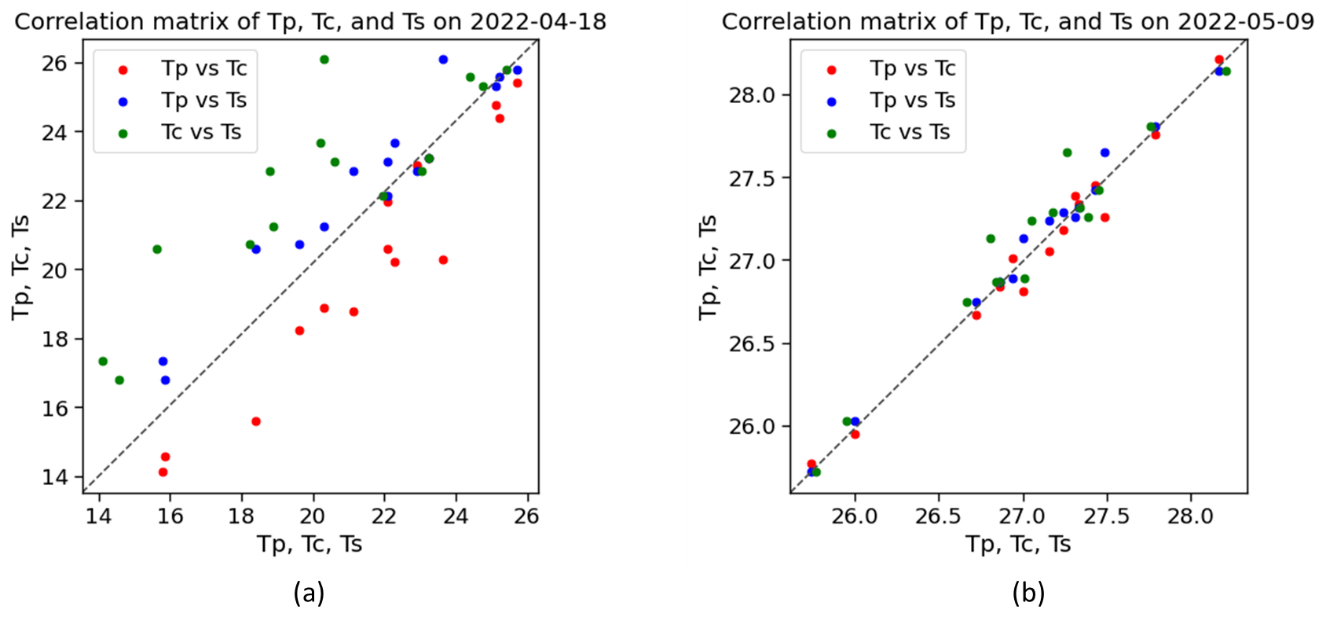


Figure S6. Scatter plots of thermal parameters in two dates of 2022.


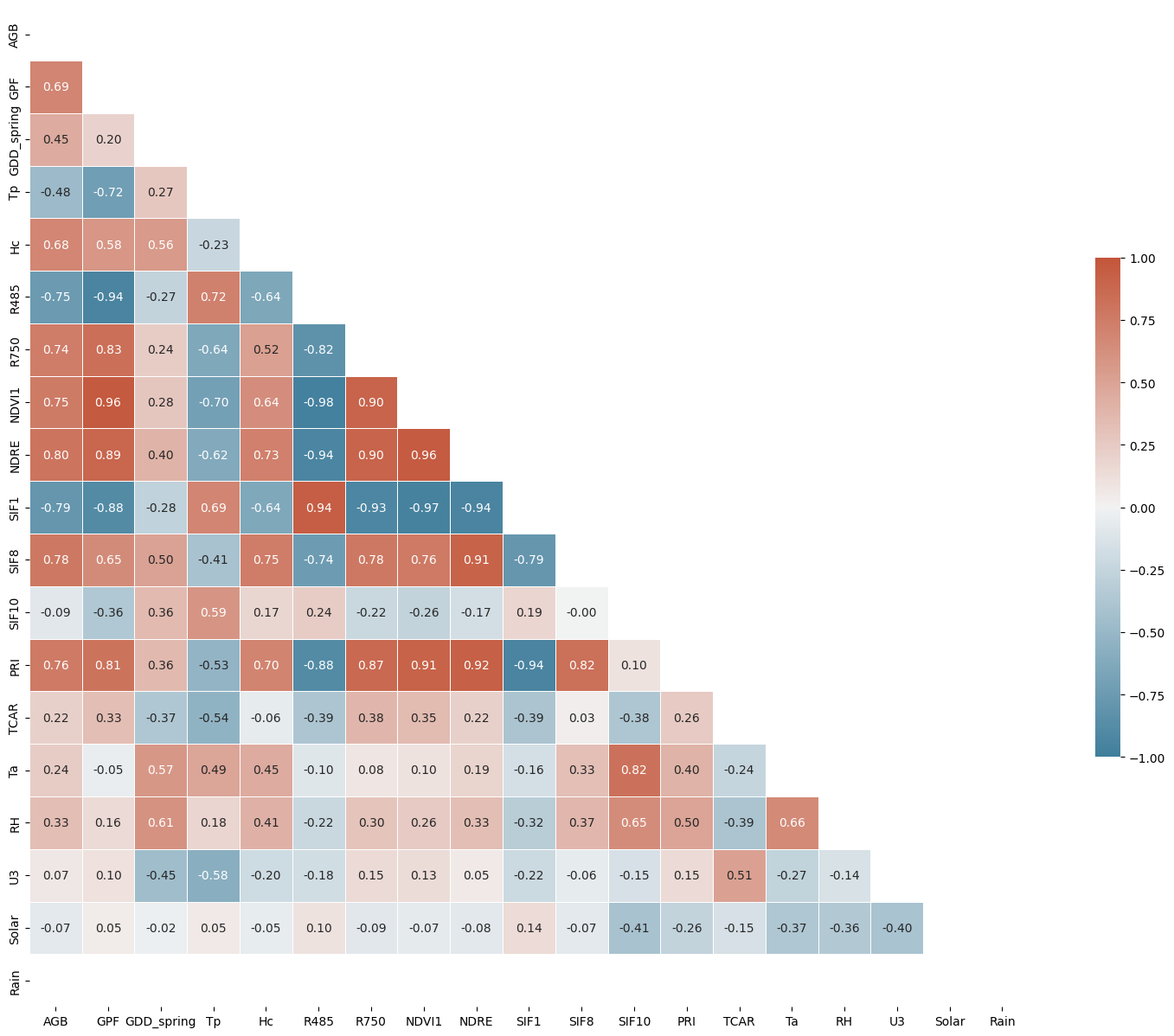


Figure S7. Correlation matrix after the first feature reduction with R values.

Table S1 Equations of Vegetation Index used in this study.

| **VI name** | **Equation** | **VI name** | **Equation** |
| --- | --- | --- | --- |
| NDVI1 | $\frac{R770-R660}{R770+R660}$ | SIF5 | $\frac{{R685}^{2}}{(R675*R690)}$ |
| NDVI2 | $\frac{R750-R705}{R750+R705}$ | SIF6 | $\frac{R685}{R655}$ |
| NDVI3 | $\frac{R800-R670}{R800+R670}$ | SIF7 | $\frac{R690}{R600}$ |
| NDRE | $\frac{R770-R660}{R770+R660}$ | SIF8 | $\frac{R740}{R800}$ |
| OSAVI | $\left( 1+0.16 \right)*\frac{R800-R670}{R800+R670}$ | SIF9 | $\frac{{R683}^{2}}{(R675*R691)}$ |
| SIF1 | $\frac{R680}{R630}$ | SIF10 | $R760.59-R759.5$ |
| SIF2 | $\frac{R685}{R630}$ | PRI | $\frac{R531-R570}{R531+R570}$ |
| SIF3 | $\frac{R687}{R630}$ | NIRv | $\frac{R800-R670}{R800+R670}*R800$ |
| SIF4 | $\frac{R690}{R630}$ | TCAR | $\frac{3*[\left( R700-R670 \right)-0.2\left( R700-R550 \right)*\left( \frac{R700}{R670} \right)]}{\left( 1+0.16 \right)*(R800-R670)/(R800+R670+0.16)}$ |

| $GDD= \frac{T_{max}+T_{min}}{2}- T_{cutoff}$ | Equation S1 |
| --- | --- |

Where GDD is Growing Degree Day that equals to 0 if the calculated GDD is negative; T_max_ is daily maximum air temperature in Celsius; T_min_ is daily minimum air temperature in Celsius; T_cutoff_ is the cutoff temperature in Celsius.

Table S2. Ranges of hyperparameters used in the grid search for the optimal models in the four different machine learning algorithms.

| **Model** | **Parameter** | **Value ranges used in cross validation** |
| --- | --- | --- |
| Radom Forests  (RF) | Number of trees | 100 - 1000 |
|  | Maximum depth of a tree | 1 - 12 |
|  | Minimum sample to split | 2 - 10 |
|  | Minimum samples on the leaf | 1 - 5 |
|  | Maximum feature count | ‘auto’, ‘sqrt’, ‘log2’ |
|  | Bootstrap | True, False |
| Support Vector Regression  (SVR) | C | 0.001, 0.01, 0.1, 1, 10, 100, 1000, 10000 |
|  | epsilon | 0.001, 0.01, 0.1, 1, 10, 100 |
|  | kernel | 'linear', 'poly', 'rbf', 'sigmoid' |
|  | degree | 2, 3, 4, 5 |
|  | gamma | ‘scale’, ‘auto’ |
| Partial Least Squares Regression  (PLSR) | Number of components | 1 to 12 |
| Artificial Neuron Network  (ANN) | epochs | 300, 400, 500 |
|  | Learning rate | 0.0001, 0.001, 0.01, 0.1 |
|  | Neuron count of hidden layer 1 | 5, 10, 15, 20, 25 |
|  | Neuron count of hidden layer 2 | 5, 10, 15, 20, 25 |
|  | Regularization of hidden layer 1 | 0, 0.5, 1 |
|  | Regularization of hidden layer 2 | 0, 0.5, 1 |

Table S3. Best model parameters of the investigated machine learning models using grid search.

| **Model name** | **Parameter** | **Value** | **Model name** | **Parameter** | **Value** |
| --- | --- | --- | --- | --- | --- |
| Radom Forests  (RF) | Number of estimators | 600 | Partial Least Squares Regression  (PLSR) | Number of components | 14 |
|  | Maximum depth | 8 | Artificial Neuron Network  (ANN) | epochs | 500 |
|  | Minimum sample to split | 5 |  | Learning rate | 0.01 |
|  | Minimum samples on the leaf | 5 |  |  |  |
|  | Maximum feature count | ‘auto’ |  | Neuron count of hidden layer 1 | 25 |
| Support Vector Regression  (SVR) | C | 1000 |  | Neuron count of hidden layer 2 | 5 |
|  | gamma | ‘scale’ |  | Regularization of hidden layer 1 | 0 |
|  | kernel | 'linear' |  | Regularization of hidden layer 2 | 0 |
|  | degree | 2 |  |  |  |
